# Egg White Hydrolysate Mitigates Cadmium-induced Neurological Disorders and Oxidative Damage

**DOI:** 10.1007/s11064-024-04110-2

**Published:** 2024-02-14

**Authors:** José Eudes Gomes Pinheiro Júnior, Priscila Marques Sosa, Ben-Hur Souto das Neves, Dalton Valentim Vassallo, Franck Maciel Peçanha, Marta Miguel-Castro, Pâmela Billig Mello-Carpes, Giulia Alessandra Wiggers

**Affiliations:** 1 Graduate Program in Biochemistry and Multicentric Graduate Program in Physiological Sciences, Universidade Federal do Pampa, BR 472 – km 592, Uruguaiana, 97500-970 Rio Grande do Sul Brazil; 2 Departments of Physiological Sciences, Universidade Federal do Espírito Santo and School of Medicine of Santa Casa de Misericórdia (EMESCAM), Av. Marechal Campos 1468, Vitória, 29040-090 Espírito Santo Brazil; 3 Bioactivity and Food Analysis Laboratory, Instituto de Investigación en Ciencias de la Alimentación, Nicolás Cabrera, 9, Campus Universitario de Cantoblanco, Madrid, 28049 Spain

**Keywords:** Anxiety, Cadmium, Egg white hydrolysate, Neurological deficits, Oxidative stress

## Abstract

We aimed to investigate whether the consumption of Egg White Hydrolysate (EWH) acts on nervous system disorders induced by exposure to Cadmium (Cd) in rats. Male Wistar rats were divided into (a) Control (Ct): H_2_O by gavage for 28 days + H_2_O (i.p. − 15th − 28th day); (b) Cadmium (Cd): H_2_O by gavage + CdCl_2_ − 1 mg/kg/day (i.p. − 15th − 28th day); (c) EWH 14d: EWH 1 g/kg/day by gavage for 14 days + H_2_O (i.p.- 15th − 28th day); (d) Cd + EWH cotreatment (Cd + EWHco): CdCl_2_ + EWH for 14 days; (e) EWH 28d: EWH for 28 days; (f) EWHpre + Cd: EWH (1st − 28th day) + CdCl_2_ (15th − 28th day). At the beginning and the end of treatment, neuromotor performance (Neurological Deficit Scale); motor function (Rota-Rod test); ability to move and explore (Open Field test); thermal sensitivity (Hot Plate test); and state of anxiety (Elevated Maze test) were tested. The antioxidant status in the cerebral cortex and the striatum were biochemically analyzed. Cd induces anxiety, and neuromotor, and thermal sensitivity deficits. EWH consumption prevented anxiety, neuromotor deficits, and alterations in thermal sensitivity, avoiding neuromotor deficits both when the administration was performed before or during Cd exposure. Both modes of administration reduced the levels of reactive species, and the lipid peroxidation increased by Cd and improved the striatum’s antioxidant capacity. Pretreatment proved to be beneficial in preventing the reduction of SOD activity in the cortex. EWH could be used as a functional food with antioxidant properties capable of preventing neurological damage induced by Cd.

## Highlights

EWH prevented neurological damage induced by Cd exposure.

EWH prevented anxiety and neuromotor, and thermal sensitivity deficits before or during Cd exposure.

EWH improved the redox balance before or during Cd exposure.

## Introduction

Cadmium (Cd) is a heavy metal, without a highly toxic biological role defined and widely used by humans in industrial activities [1]. Its toxic effects have been demonstrated mostly in the cardiovascular [2] and the reproductive system [3]. The toxic damages caused by this metal are related to the increase of oxidative stress [4, 5], apoptosis [6, 7], autophagy [8], genetic deregulation [9, 10] and interaction with other elements such as calcium and zinc [11].

Cd can cross the blood-brain barrier (BBB) [12] and diffuse in the brain, increasing the oxidative stress and reducing antioxidant defenses [13], promoting behavioral changes [14, 15], alterations in cholinergic mechanics [16] and central and peripheral electrophysiological activity [17]. Cd poisoning has been related to amyotrophic lateral sclerosis development due to a deficiency of the brain SOD enzyme [18], increased risk of stroke [19], and neurodegenerative diseases [20].

The increase in contamination by this toxic metal has motivated research investigating the therapeutic effects of chelating and/or antioxidant compounds capable of protecting biological systems against their toxic damage [21]. Thus, functional foods such as egg white hydrolysate (EWH) have demonstrated multiple biological effects against Cd [22, 23] and other metal poisonings (Hg and Al) [24, 25]. Enzymatic hydrolysis to produce EWH results in an ingredient rich in bioactive peptides. Some beneficial effects have been reported, such as vasodilator [26], antihypertensive [27, 28], anti-inflammatory [29], angiotensin-converting enzyme inhibitor [27], and antioxidant [30–32] in different experimental models. Moreover, the EWH improved memory parameters and cognitive dysfunction of rats exposed to low doses of mercury and long-term exposure to Al, respectively [24, 32].

Herein, we investigated the protective effect of EWH against toxicity induced by high Cd concentrations in the nervous system of rats.

## Methods

### Animals and Experimental Design

Ninety-day male Wistar rats (290–330 g) were maintained at standard conditions (constant room temperature, humidity, and 12:12 h light-dark) with water and fed *ad libitum* in the Federal University of Pampa vivarium. The experimental protocols were performed according to the guidelines stated by the Brazilian Societies of Experimental Biology and National Institute of Health Guide for the Care and Use of Laboratory Animals (NIH, 1996) and the Local Institution Animal Care and Use Committee (protocol number 012/2019 and 013/2019).

**Fig. 1:**
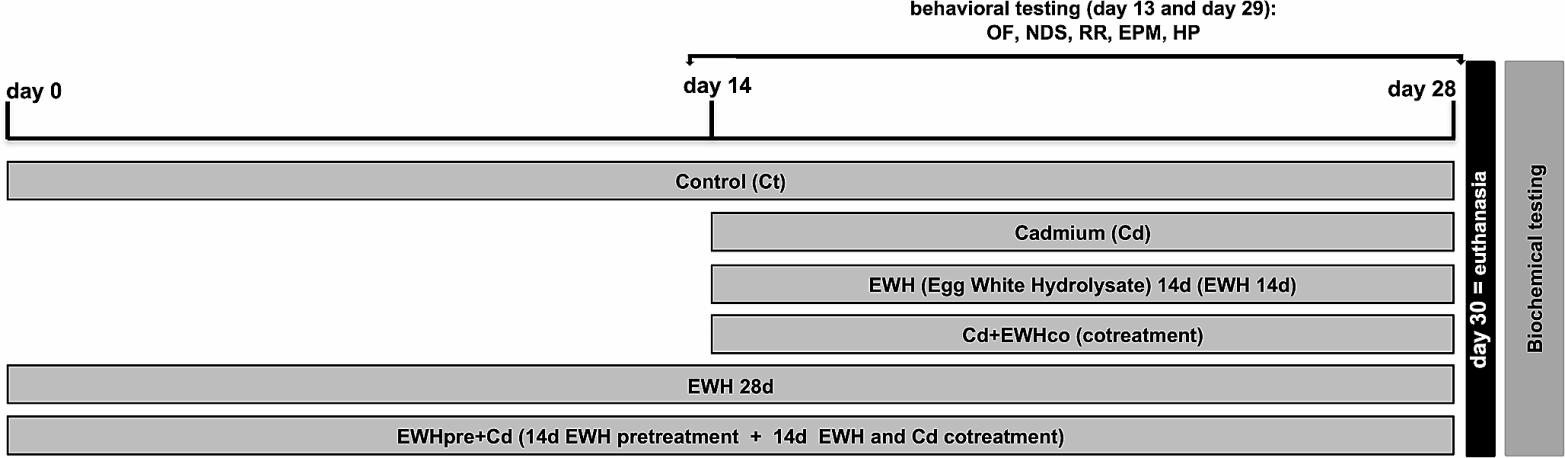
Experimental design. All rats were submitted to the Open Field (OF), Neurologic Disabilities Scale (NDS), Rota-rod (RR), Elevated Plus Maze (EPM) and Hot Plate (HP) and tests on days 13 and 29 of the protocol, equivalent to the initial and final times of Cd injection. The groups are represented in horizontal bars

Animals were randomly distributed into six groups (Fig. 1):

1. Control (Ct): drinking water by gavage + distilled water intraperitoneally (i.p.) for 28 days;
2. Cadmium (Cd): Cadmium chloride – CdCl_2_ − 1 mg/kg i.p. for 14 days [33] + drinking water by gavage;
3. EWH 14d: EWH  1 g/kg/day by gavage for 14 days + distilled water i.p [22]. ;
4. Cd + EWHco: cotreatment with CdCl_2_ 1 mg/kg i.p. + EWH 1 g/kg/day by gavage for 14 days [22];
5. EWH 28d: EWH 1 g/kg/day by gavage for 28 days + distilled water i.p.;
6. EWHpre + Cd: pretreatment with EWH 1 g/kg/day by gavage for 14 days + distilled water i.p. followed by CdCl_2_ − 1 mg/kg i.p. + EWH − 1 g/kg/day for 14 days.

The animals were submitted to all behavioral tests on days 13 and 29 of the protocol following the sequence: Open Field (OF), Neurologic Disabilities Scale (NDS), Rota-Rod (RR), Elevated Plus Maze (EPM) and Hot Plate (HP) (Fig. 1). This sequence was based on carrying out tests from lower capacity to generate stress to greater capacity to generate stress.

### EWH Preparation

The egg white hydrolysate (EWH) was prepared for the treatment according to Garcés-Rimón et al. [29]. In summary, for obtained the EWH, the hydrolysis processes used Pepsin from pork stomach (E.C. 3.4.23.1 - Cardif, United Kingdom: 1:3000; E:S: 2:100 w-w, pH 2.0, 38 °C) mixed with commercial pasteurized egg white for 8 h. The inactivation of the enzyme was achieved by increasing the pH to 7.0 with 5 N NaOH. The EWH was centrifuged (2500 g, 15 min.), and the supernatants were frozen and lyophilized. The main bioactive peptides identified in EWH were previously checked by reverse-phase liquid chromatography-mass spectrometry (RP-HPLC-MS/MS): FRADHPFL, RADHPFL, YAEERYPIL, YRGGLEPINF, ESIINF, RDILNQ, IVF, YQIGL, SALAM, FSL [30, 34].

### Behavior Tests

We evaluated the impact of Cd administration and EWH supplementation on neuromotor performance, locomotor and exploratory activity, motor coordination and balance, anxious-like behavior, and sensitivity. We performed all tests on days 13 and 29, equivalent to a pre and post-treatment evaluation (Fig. 1).

#### Neurologic Disabilities Scale (NDS)

The Neurologic Disabilities Scale (NDS), to assess neuromotor performance, was performed. The test evaluates the animal’s ability to walk on the contralateral forelimb’s beam and flexion, retraction of the contralateral hindlimb, spontaneous spins, and bilateral seizures of the front legs. The animals were allocated and received a score ranging from 0 to 4, where 4 indicates severe neurological deficit, according to Bederson et al. [35].

#### Rota-Rod (RR)

To evaluate motor coordination and balance, we used the Rota-Rod (RR) task. The apparatus consisted of a cylinder; suspended 20 cm from the device surface, driven by a gear that maintains a constant speed. First, we did habituation to the apparatus at a rate of 16 rotations per minute (rpm). After, we performed the test at 26 rpm. We placed the rats on the cylinder, and in 5 min, we recorded: (i) the time for the first fall and (ii) the number of falls [36].

#### Open Field (OF)

We assessed the exploratory and locomotor activity by open field (OF) task, as previously described by Bonini et al. [37]. The apparatus consists of a box made of wood a box (50 × 50 × 50 cm) in which black lines divide the floor into 12 equal squares. We placed the rats individually in the center of the apparatus, and during a session of five minutes, we recorded the number of crossings and rearings.

#### Hot Plate (HP)

To analyze the sensitivity/pain threshold of the rats, we used an HP test. The apparatus consists of a plate enclosed by a 25 cm high, lidded perspex box measuring 145 × 275 mm. The test consisted of placing a rat on a metal platform. This platform is progressively heated. We recorded the time that the rats led to withdrawing their paws. To avoid injuries in the paws, we imposed a ceiling of 60 s [38].

#### Elevated plus Maze (EPM)

The Elevated Plus Maze test (EPM) was used to evaluate the anxiety behavior; each rat was placed in the maze, which contains two open and two closed arms, for 5 min, and the time in the open arms of the maze was monitored for each rat as previously described [39].

### Biochemicals Tests

At the end of treatments and behavior testing, rats were euthanized, and the cortex and striatum from the brain were quickly dissected and homogenized (50mM Tris HCl, pH 7.4, 1/10, w/v), centrifuged (2400 g, 10 min, 4 °C) and frozen at − 80 °C for performed oxidative stress biochemical analysis. Levels of reactive oxygen species (ROS) were determined by the spectrofluorometric method described by Loetchutinat et al. [40]. The ROS levels were expressed as fluorescence units (FU). Lipid peroxidation was measured as malondialdehyde (MDA) using a colorimetric method, as previously described by Ohkawa et al. [41], with modifications [42], and results were expressed as nmol of MDA/mg of protein. The total antioxidant capacity in the cortex and striatum tissue was measured by Ferric Reducing Antioxidant Power (FRAP) Assay [43], with modifications [42] and results were presented with particular reference to Trolox equivalents, Trolox (µmol/mL). Superoxide dismutase (SOD) activity was assayed using spectrophotometry, as described by Misra and Fridovich [44], and expressed as units (U)/mg of protein.

According to the Bradford method [45], the protein was measured using bovine serum albumin as a standard. All reagents and salts were obtained from Sigma-Aldrich and Merck (Darmstadt, Germany).

### Statistical Analysis

Results are expressed as mean ± SD. Data from behavior tests were analyzed using two-way ANOVA to assess the effects of different treatments over time (time factor) and to assess the effects of different treatments between groups (treatment factor), as well as the interaction between the two factors (time x treatment factors). Data from biochemical tests were analyzed using two-way ANOVA to assess the effects of Cadmium administration (Cd factor) and to assess the effects of EWH between groups (treatment factor), as well as the interaction between the two factors (Cd x EWH factors). When two-way ANOVA showed a statistically significant difference in any of these factors, or interaction between them, a Bonferroni’s *post-hoc* analysis was performed. Differences were considered statistically significant when *P* < 0.05.

## Results

### Behavioral Tests

#### Neurological Disabilities Scale (NDS)

In the NDS, two-way ANOVA revealed an effect over time (F _(1, 52)_ = 6.834, *P* = 0.0117) and an effect of the treatment (F _(5, 52)_ = 8.185, *P* < 0.001). There was an interaction between both factors (F _(5,52)_ = 8.185, *P* = 0.0001).

*Post-hoc* analyses showed that the neuromotor performance did not alter over time in the Ct group (t _(52)_ = 0.000, *P* > 0.999 for 0 vs. 14th day, Table 1). On the other hand, cadmium administration for 14 days induced a neurological deficit (t _(52)_ = 7.466, *P* < 0.0001, Table 1). EWH supplementation for 14 days did not affect neurological performance over time (t _(52)_ = 0.000, *P* > 0.999 for the EWH14d group). In the same way, EWH administration for 28 days did not induce the neurological deficit (t _(52)_ = 0.000, *P* > 0.999 for EWH28d, Table 1). Both Cd + EWHco (t = 0.519, *P* > 0.9999, Table 1) and EWHpre + Cd (t = 0.000, *P* > 0.999, Table 1) groups did not show neurological deficits over time.

**Table 1:** Effects of Egg White Hydrolysate (EWH) administration and Cd on neuromotor function evaluated by Neurological Deficit Scale (NDS).

Groups		Ct	Cd	EWH 14d	Cd + EWHco	EWH 28d	EWHpre + Cd
N		10	14	10	10	5	9
Score (Mean)	Day 13	0.000	0.000	0.000	0.000	0.000	0.000
	Day 29	0.000	1.214*	0.000	0.1000	0.000	0.000

* vs. day 0. EWHco = cotreatment; EWHpre = pretreatment

When we compare groups, animals from all groups did not express neurological deficit on day 0 (*P* > 0.05 for all groups, Table 1). On the 14th day, animals from the Cd group showed a neurological deficit compared to the Ct group (t _(104)_ = 6.816, *P* < 0.0001, Table 1). Animals from both groups Cd + EWHco (t _(104)_ = 6.254, *P* < 0.0001) and EWHpre + Cd (t _(104) =_ 6.605, *P* < 0.0001) did not show neurological deficit compared to the Cd group.

#### Rota-Rod (RR) Task

On the RR test, two-way ANOVA did not reveal an effect on the number of falls over time (F _(1, 49)_ = 0.242, *P* = 0.622) but revealed an effect of the treatment (F _(5, 49)_ = 2.673, *P* < 0.032).

**Fig. 2:**
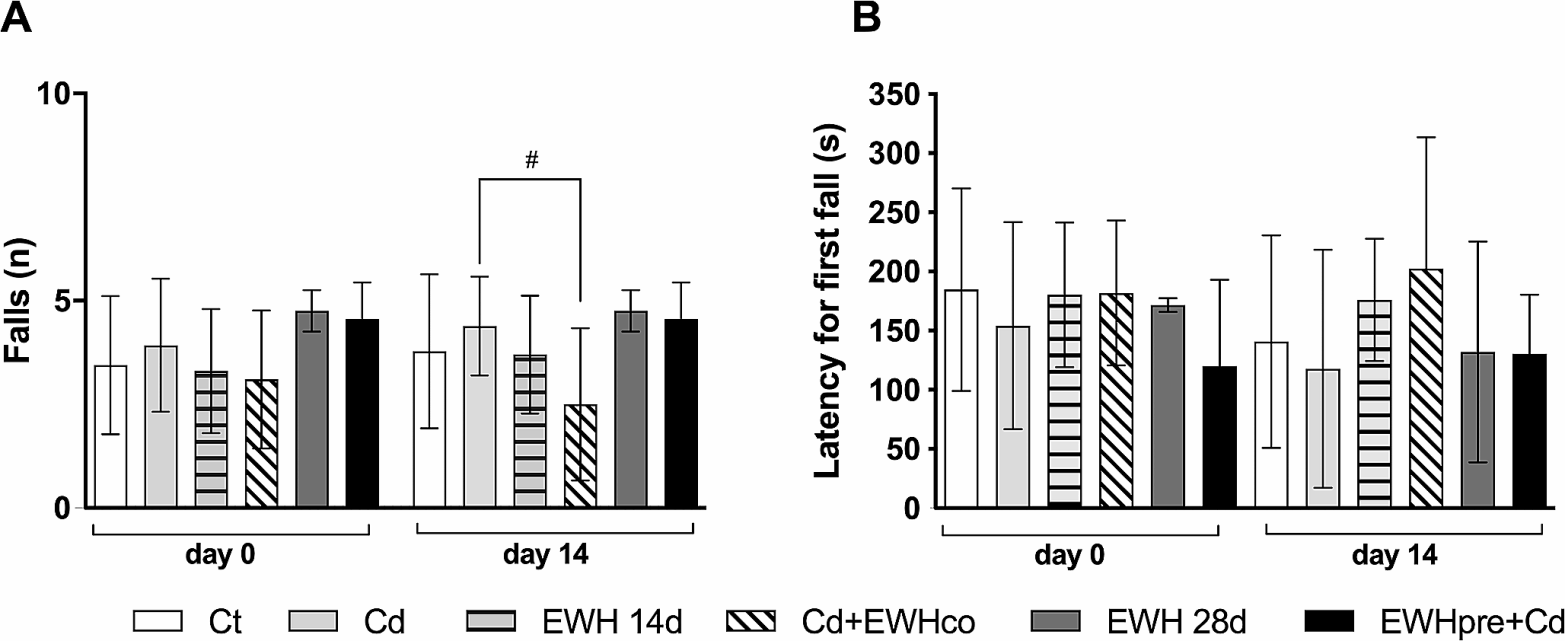
Effects of EWH on motor coordination and balance evaluated by Rota-Rod (RR) in Cd-exposure rats. (**A**) The number of falls. (**B**) Latency to the first fall, in seconds. Data are presented as the mean ± SD. ^#^*P* < 0.05 in the groups’ comparison (*n* = 5–13). Ct: drinking water by gavage + distilled water intraperitoneally (i.p.) for 28 days; Cd: Cadmium chloride – CdCl_2_ − 1 mg/kg i.p. for 14 days + drinking water by gavage; EWH 14d: egg white hydrolysate 1 g/kg/day by gavage for 14 days + distilled water i.p.; Cd + EWHco: cotreatment with CdCl_2_ 1 mg/kg i.p. + EWH 1 g/kg/day by gavage for 14 days; EWH 28d: egg white hydrolysate 1 g/kg/day by gavage for 28 days + distilled water i.p.; EWHpre + Cd: pretreatment with EWH 1 g/kg/day by gavage for 14 days + distilled water i.p. followed by CdCl_2_ − 1 mg/kg i.p. + EWH − 1 g/kg/day for 14 days

There was no change in the number of falls over time (*P* > 0.05 for all groups, Fig. 2A). In the same way, there was no change in the number of falls when comparing the groups on day 0 (*P* > 0.05 for all groups, Fig. 2A). On the 14th day, the Cd group showed no change in the number of falls compared to the Ct group (t _(98)_ = 0.9668, *P* > 0.999, Fig. 2A). However, there was a reduction in the number of falls in animals in the Cd + EWHco group compared to animals in the Cd group (t _(98)_ = 3.095, *P* = 0.0384, Fig. 2A).

Regarding the latency for the first fall, two-way ANOVA revealed no effect over time (F _(1, 48)_ = 1.086, *P* = 0.3026), nor effect of the treatment between the groups (F _(5, 48)_ = 1.508, *P* = 0.2049). There was no interaction between factors (F _(5, 48)_ = 0.7308, *P* = 0.6038) (Fig. 2B).

#### Open Field (OF)

On the OF, two-way ANOVA revealed an effect on the crossing over time (F _(1, 52)_ = 51.56, *P* < 0.0001) and no effect of the treatment (F _(5, 52)_ = 4.097, *P* = 0.0033). There was no interaction between factors (F _(5, 52)_ = 1.704, *P* = 0.1501).

**Fig. 3:**
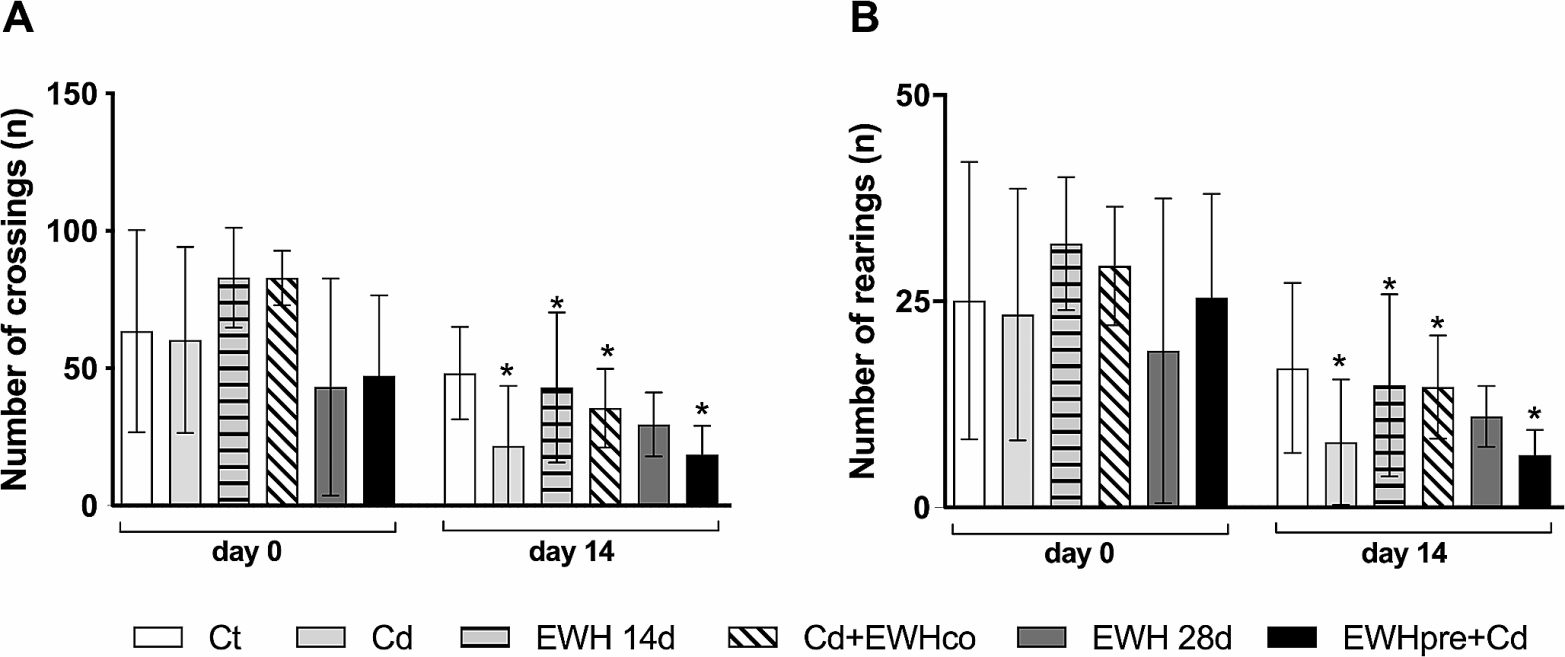
Effects of Egg White Hydrolysate (EWH) on locomotor and exploratory activities evaluated by Open Field (OF) in Cd-exposure rats. (**A**) The number of crossings. (**B**) The number of rearings. Data are presented as the mean ± SD. ^*^P < 0.05 vs. day 0 (*n* = 5–13). Ct: drinking water by gavage + distilled water intraperitoneally (i.p.) for 28 days; Cd: Cadmium chloride – CdCl_2_ − 1 mg/kg i.p. for 14 days + drinking water by gavage; EWH 14d: egg white hydrolysate 1 g/kg/day by gavage for 14 days + distilled water i.p.; Cd + EWHco: cotreatment with CdCl_2_ 1 mg/kg i.p. + EWH 1 g/kg/day by gavage for 14 days; EWH 28d: egg white hydrolysate 1 g/kg/day by gavage for 28 days + distilled water i.p.; EWHpre + Cd: pretreatment with EWH 1 g/kg/day by gavage for 14 days + distilled water i.p. followed by CdCl_2_ − 1 mg/kg i.p. + EWH − 1 g/kg/day for 14 days

Animals from the Ct group showed no change in locomotor activity over time (t _(52)_ = 1.563, *P* = 0.7449; Fig. 3A). However, administration of Cd for 14 days led to a reduction in locomotor activity over time (t _(52)_ = 4.662, *P* = 0.0001; Fig. 3A). Surprisingly, the supplementation with EWH for 14 days reduced locomotor activity (t _(52)_ = 4.086, *P* = 0.0009; Fig. 3A). Cotreatment with EWH for 14 days and pre-administration for 28 days were not able to prevent the impairment of locomotor activity induced by Cd (t _(52)_ = 4.842, *P* < 0.0001 for Cd + EWHco; t _(52)_ = 2.789, *P* = 0.0443 for EWHpre + Cd; Fig. 3A). EWH supplementation for 28 days did not affect locomotor activity per se (t _(52)_ = 0.9823, *P* > 0.999 for EWH28d; Fig. 3A).

There was no statistically significant difference between the groups in the number of crossing days 0 or 14 (*P* > 0.05 for all groups; Fig. 3A).

We also assessed exploratory activity by counting the number of rearing. Two-way ANOVA revealed an effect over time (F _(1, 52)_ = 41.73, *P* = 0.0001) but not an effect of the treatment (F _(5, 52)_ = 2.092, *P* = 0.0815). There was no interaction between factors (F _(5, 52)_ = 0.7412, *P* = 0.5962).

In the control condition, there was no change in exploratory activity over time (t _(51)_ = 1.681, *P* = 0.593; Fig. 3B). Animals from the Cd groups showed a reduction in exploratory activity (t _(51)_ = 3.613, *P* = 0.004; Fig. 3B). Supplementation with EWH for 14 days also reduced exploratory activity (t _(51)_ = 3.525, *P* = 0.0054; Fig. 3B). We observed similar results in both groups Cd + EWHco (t _(51)_ = 3.525, *P* = 0.024; Fig. 3B) and EWHpre + Cd (t _(51)_ = 3.716; *P* = 0.003; Fig. 3B). EWH supplementation for 28 days did not affect locomotor activity (t _(51)_ = 1.159, *P* > 0.999; Fig. 3B). There was no statistically significant difference between the groups in the number of rearing on days 0 and 14 (*P* > 0.05 for all groups; Fig. 3B).

#### Hot Plate (HP)

On the HP test, two-way ANOVA revealed an effect over time (F _(1, 52)_ = 4.532, *P* = 0.038) but not an effect of the treatment (F _(5, 52)_ = 1.699, *P* = 0.151). There was an interaction between two factors (F _(5, 52)_ = 2.577, *P* = 0.0371).

**Fig. 4:**
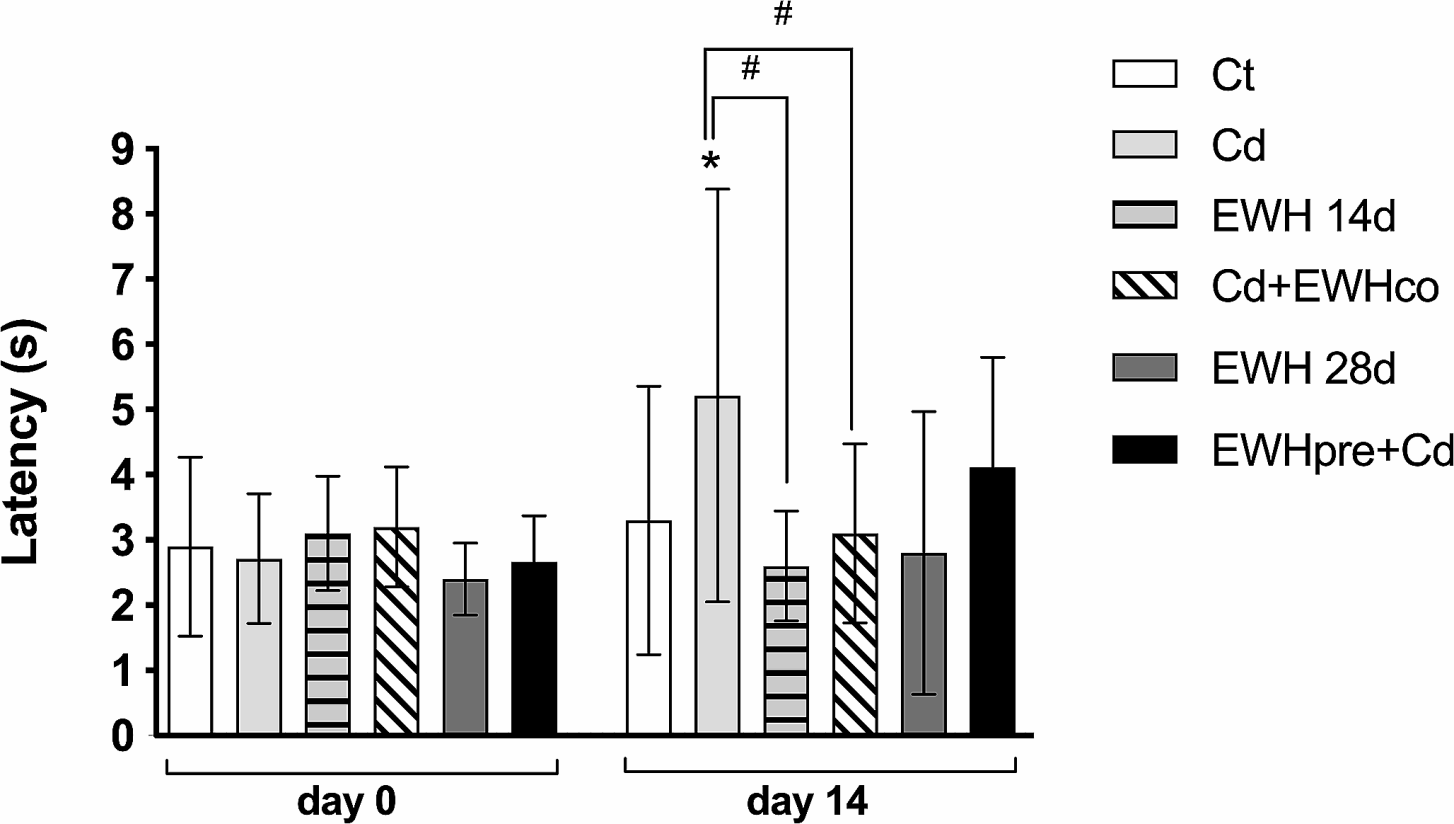
Effects of Egg White Hydrolysate (EWH) on peripherical thermal sensibility evaluated by Hot Plate (HP) in Cd-exposure rats (latency to paw withdraw). Data are presented as the mean ± SD. ^*^P < 0.05 vs. day 0; ^#^*P* < 0.05 for comparison between groups (*n* = 5–14). Ct: drinking water by gavage + distilled water intraperitoneally (i.p.) for 28 days; Cd: Cadmium chloride – CdCl_2_ − 1 mg/kg i.p. for 14 days + drinking water by gavage; EWH 14d: egg white hydrolysate 1 g/kg/day by gavage for 14 days + distilled water i.p.; Cd + EWHco: cotreatment with CdCl_2_ 1 mg/kg i.p. + EWH 1 g/kg/day by gavage for 14 days; EWH 28d: egg white hydrolysate 1 g/kg/day by gavage for 28 days + distilled water i.p.; EWHpre + Cd: pretreatment with EWH 1 g/kg/day by gavage for 14 days + distilled water i.p. followed by CdCl2 − 1 mg/kg i.p. + EWH − 1 g/kg/day for 14 days

Ct group rats showed no change in peripheral thermal sensitivity over time (t _(52)_ = 0.5368, *P* > 0.999, Fig. 4). Cd administration increased the latency in the withdrawal of the paws (t _(52)_ = 3.970, *P* = 0.001, Fig. 4). Animals from both groups Cd + EWHco (t _(52)_ = 0.134, *P* > 0.999, Fig. 4) and EWHpre + Cd (t _(52)_ = 1.839, *P* = 0.429, Fig. 4) did not show alteration in the latency of withdrawal of the paws over time.

When comparing the groups, animals from all groups did not express alteration in the latency of withdrawal of the paws on day 0 (*P* > 0.05 for all groups, Fig. 4). On the 14th day, animals from the Cd group did not demonstrate increased paws’ withdrawal compared with the Ct group (t _(104)_ = 0.2720, *P* < 0.090, Fig. 4). Animals from the EWH14d group showed a decrease in the latency of the paws’ withdrawal compared with the Cd group (t _(104)_ = 0.5648, *P* = 0.0033, Fig. 4). In the same way, animals from the Cd + EWHco showed a decrease in the latency of the paws’ withdrawal compared with the Cd group (t _(104)_ = 0.7113, *P* = 0.0378).

#### Elevated plus Maze (EPM)

Regarding entries in the close arms on the EPM test, two-way ANOVA revealed an effect over time (F _(1, 52)_ = 17.65, *P* = 0.0001) and the effect of the treatment (F _(5, 52)_ = 2.998, *P* = 0.0188). There was an interaction between two factors (F _(5, 52)_ = 2.789, *P* = 0.0264).

**Fig. 5:**
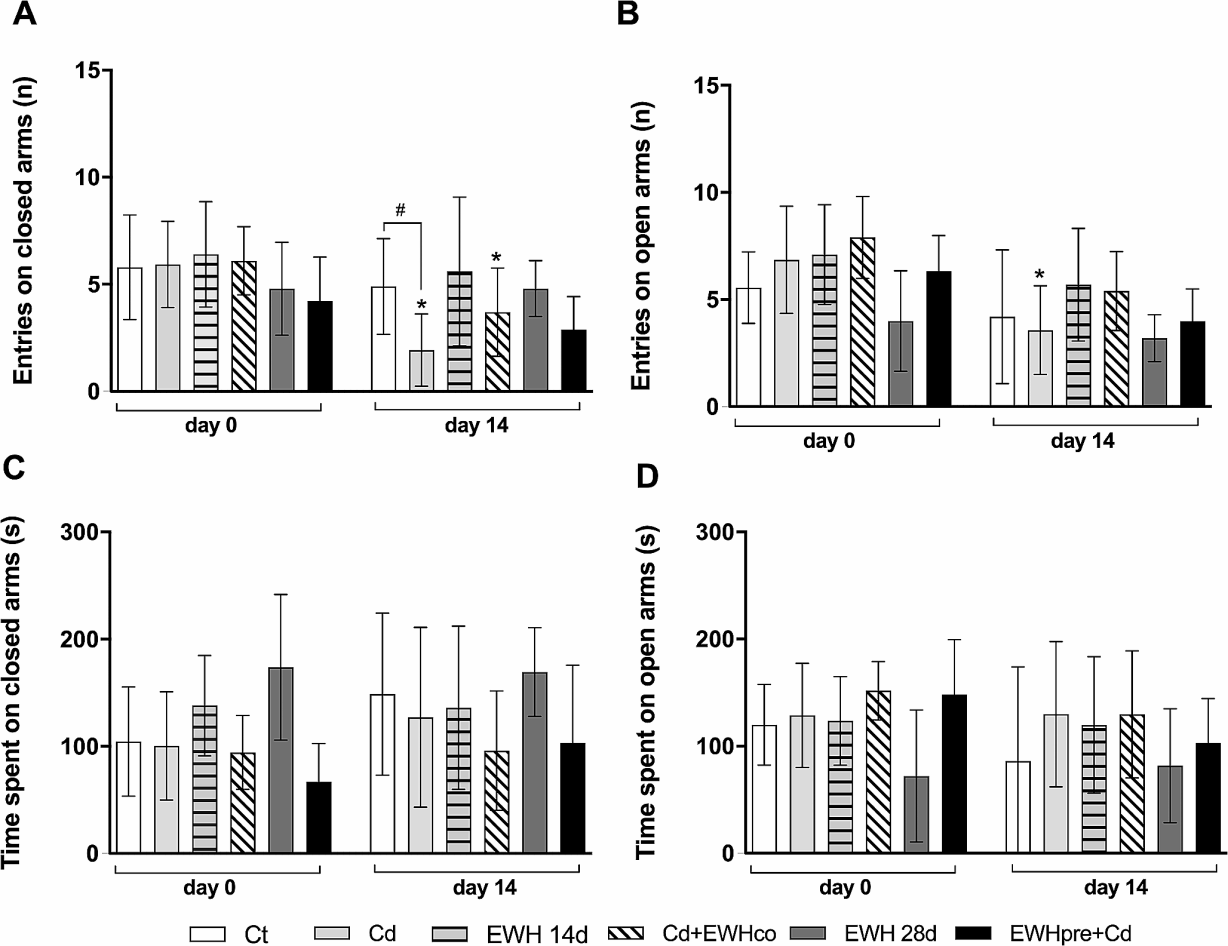
Effects of Egg White Hydrolysate (EWH) on anxiety levels by Elevate Plus Maze (EPM) in Cd-exposure rats. (**A**) Entries on closed arms. (**B**) Entries on open arms. (**C**) Time spent on closed arms. (**D**) Time spent on open arms. ^*^P < 0.05 vs. day 0; ^#^*P* < 0.05 in the groups’ comparison (*n* = 9). Ct: drinking water by gavage + distilled water intraperitoneally (i.p.) for 28 days; Cd: Cadmium chloride – CdCl_2_ − 1 mg/kg i.p. for 14 days + drinking water by gavage; EWH 14d: egg white hydrolysate 1 g/kg/day by gavage for 14 days + distilled water i.p.; Cd + EWHco: cotreatment with CdCl_2_ 1 mg/kg i.p. + EWH 1 g/kg/day by gavage for 14 days; EWH 28d: egg white hydrolysate 1 g/kg/day by gavage for 28 days + distilled water i.p.; EWHpre + Cd: pretreatment with EWH 1 g/kg/day by gavage for 14 days + distilled water i.p. followed by CdCl_2_ − 1 mg/kg i.p. + EWH − 1 g/kg/day for 14 days

The Ct group did not show a change in the number of entries in the closed arms over time (t _(52)_ = 1.036, *P* > 0.999, Fig. 5A). Cd administration decreased the number of entries in the closed arms on the 14th day when we compared it to day 0 (t _(52)_ = 5.450, *P* < 0.0001, Fig. 5A). The EWH for 14 days was not able to change the number of entries on the closed arms (t _(52)_ = 1.036, *P* > 0.9999, Fig. 5A). However, the Cd + EWHco group showed a decrease in the number of entries in the closed arms (t _(52)_ = 2.763, *P* > 0.0473, Fig. 5A). The EWH 28d group showed no change in the number of entries on the closed arms (t _(52)_ = 0.000, *P* > 0.9999, Fig. 5A). Furthermore, the EWHpre + Cd (t _(52)_ = 1.456, *P* = 0.9076, Fig. 5A) did not show alteration in the number of entries in the closed arms.

When we compared the groups, animals from all groups did not express alteration in the number of entries in the closed arms on day 0 (*P* > 0.05 for all groups, Fig. 5A). On the 14th day, animals from the Cd group demonstrated a decrease in the number of entries in the closed arms compared with the Ct group (t _(104)_ = 0.1424, *P* = 0,0204, Fig. 5A). Animals from the EWH14d group showed no change in the number of entries in the closed arms compared with the Ct group (t _(104)_ = 0.7180, *P* > 0.9999, Fig. 5A). The animals from the Cd + EWHco did not show a change in the number of entries in the closed arms compared with the Cd group (t _(104)_ = 1.962, *P* = 0.7857, Fig. 5A). In the same way, the EWHpre + Cd group did not change in the number of entries in the closed arms when we compared it with the Cd group (t _(104)_ = 1.031, *P* > 0.9999, Fig. 5A).

We assessed the number of entries in the opened arms on the EPM test, two-way ANOVA revealed an effect over time (F _(1, 52)_ = 22.92, *P* < 0.0001) and the effect of the treatment (F _(5, 52)_ = 3.595, *P* = 0.0072). However, there was no interaction between the two factors (F _(5, 52)_ = 0.9619, *P* = 0.4497) (Fig. 5B).

The Ct group showed no change in the number of entries in the opened arms over time (t _(52)_ = 1.400, *P* > 0.999, Fig. 5B). Cd administration decreased the number of entries in the opened arms on the 14th day when we compared it to day 0 (t _(52)_ = 4.185, *P* < 0.0007, Fig. 5B). On the other hand, the EWH14d group (t _(52)_ = 1.507, *P* = 0.8269, Fig. 5B), Cd + EWHco group (t _(52)_ = 2.691, *P* = 0.0573, Fig. 5B), EWH 28d group (t _(52)_ = 0.6090, *P* > 0.999, Fig. 5B), and EWHpre + Cd group (t _(52)_ = 2.383, *P* = 0.1251, Fig. 5B) did not show a change in the number of entries in the opened arms over the time.

The *post-hoc* analyses did not show a statistically significant difference in the number of entries in the opened arms when we compared the groups on day 0 (*P* > 0.05 for all groups, Fig. 5B). Likewise, there was no statistically significant difference in the number of entries into the opened arms when we compared between groups on day 14th (*P* > 0.05 for all groups, Fig. 5B).

Regarding the time spent in the closed arms, two-way ANOVA revealed no effect over time (F _(1, 51)_ = 1.771, *P* = 0.1892); however, there was an effect of the treatment (F _(5, 52)_ = 3.595, *P* = 0.0072). There was no interaction between the two factors (F _(5, 51)_ = 0.4633, *P* = 0.8017).

There was no statistically significant difference in the time spent in the closed arms on the 14th day when we compared it with day 0 (*P* > 0.05 for all groups, Fig. 5C) in the *post-hoc* test. Despite the ANOVA analysis has shown an effect of the treatment between the groups, the *post-hoc* test showed no statistically significant difference in the time spent in the closed arms neither on day 0 (*P* > 0.05 for all groups, Fig. 5C) nor on day 14th (*P* > 0.05 for all groups, Fig. 5C).

In addition, two-way ANOVA did not reveal an effect on the time spent in the open arms over time (F _(1, 52)_ = 1.919, *P* = 0.1719); however, there was an effect of the treatment (F _(5, 52)_ = 2.502, *P* = 0.0419). There was no interaction between the two factors (F _(5, 51)_ = 0.5919, *P* = 0.7062).

There was no statistically significant difference in the time spent in the opened arms on the 14th day when we compared it with day 0 (*P* > 0.05 for all groups, Fig. 5D) in the *post-hoc* test. Despite the ANOVA analysis has shown an effect of the treatment between the groups, the *post-hoc* test showed no statistically significant difference in the time spent in the open arms neither on day 0 (*P* > 0.05 for all groups, Fig. 5C) nor on day 14th (*P* > 0.05 for all groups, Fig. 5D)

### Biochemical Results

Biochemical analysis corroborates our behavioral findings. On ROS levels, two-way ANOVA revealed an effect of Cd (F _(1, 31)_ = 43.40, *P* < 0.0001), an effect of the treatment with EWH (F _(2, 31)_ = 4.559, *P* = 0.00184), and an interaction between factors (F _(2, 31)_ = 11.06, *P* = 0.0002).

**Fig. 6:**
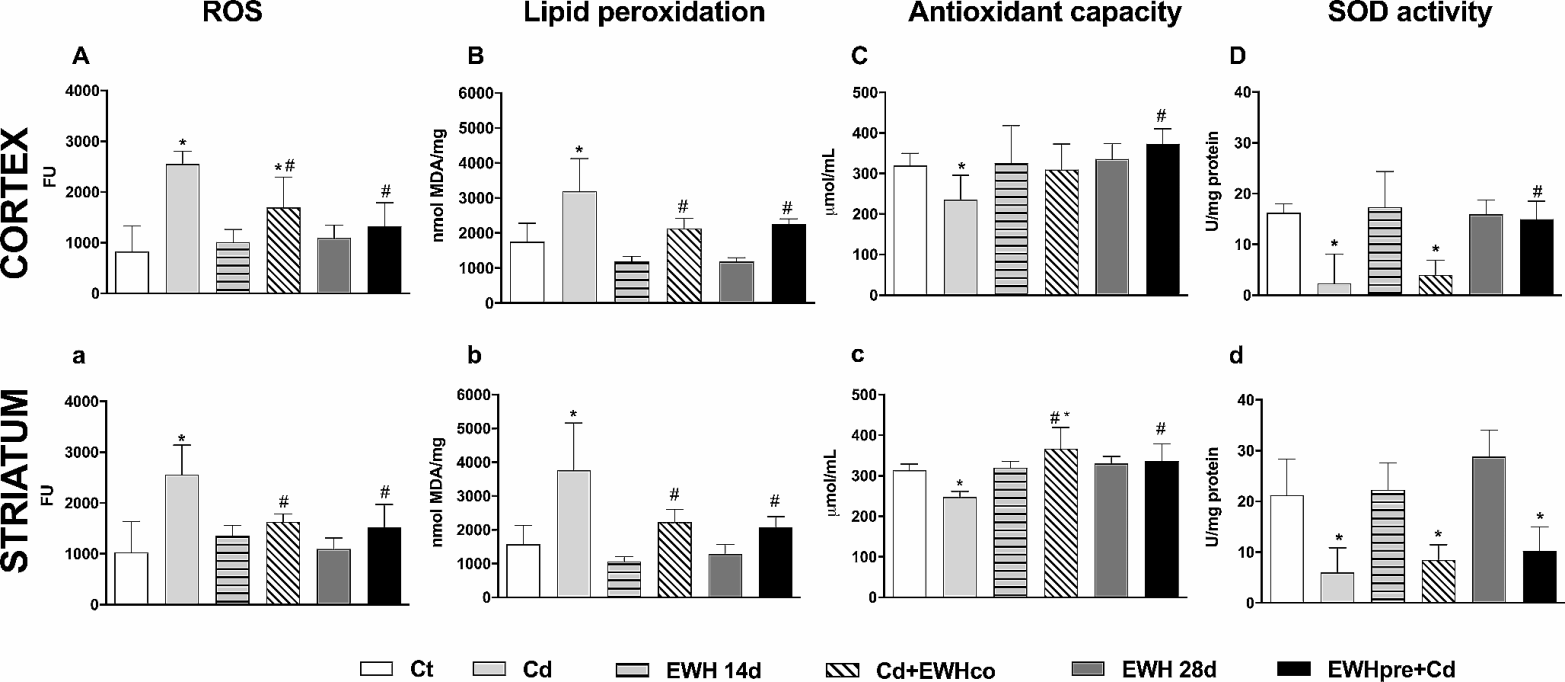
Effect of Egg White Hydrolysate (EWH) on oxidative stress in Cd-exposure rats. (**A** and **a**) Reactive oxygen species (ROS) levels expressed in fluorescence units (FU). (**B** and **b**) lipoperoxidation by Thiobarbituric Acid Reactive Substances (TBARS) expressed as nmol MDA/mg protein. (**C** and **c**) Total antioxidant capacity by Ferric Reducing Antioxidant Power Assay (FRAP) expressed as Trolox (µmol/mL). (**D** and **d**) Superoxide dismutase activity as units (U)/mg of protein. Capital letters indicate the measurements in the cortex, and lower-case in the striatum. Data are presented as the mean ± SD. *P* < 0.05 * vs. Ct group; ^#^ vs. Cd group (ANOVA followed by Bonferroni *post-hoc* test) (*n* = 8). Ct: drinking water by gavage + distilled water intraperitoneally (i.p.) for 28 days; Cd: Cadmium chloride – CdCl_2_ − 1 mg/kg i.p. for 14 days + drinking water by gavage; EWH 14d: egg white hydrolysate 1 g/kg/day by gavage for 14 days + distilled water i.p.; Cd + EWHco: cotreatment with CdCl_2_ 1 mg/kg i.p. + EWH 1 g/kg/day by gavage for 14 days; EWH 28d: egg white hydrolysate 1 g/kg/day by gavage for 28 days + distilled water i.p.; EWHpre + Cd: pretreatment with EWH 1 g/kg/day by gavage for 14 days + distilled water i.p. followed by CdCl_2_ − 1 mg/kg i.p. + EWH − 1 g/kg/day for 14 days

Animals exposed to Cd increased the ROS levels in the cortex (t _(31)_ = 7.626, *P* < 0.0001; Fig. 6A) and striatum (t _(31)_ = 6.555, *P* < 0.0001; Fig. 6a). However, EWH pretreatment (EWHpre + Cd) (cortex: t _(31)_ = 5.400, *P* = 0.0001, Fig. 6A; striatum: t _(31)_ = 4.417, *P* = 0.0017, Fig. 6a) and cotreatment (Cd + EWHco) (cortex: t _(31)_ = 3.776, *P* = 0.0102, Fig. 6A; striatum: t _(31)_ = 3.984, *P* = 0.0057, Fig. 6a) significantly reduced the ROS levels in both cerebral areas investigated. ROS levels in Cd + EWHco remain elevated when compared to the cortex of control rats (t _(31)_ = 3.710, *P* = 0.0122; Fig. 6A). There was no statistically significant difference between Ct, EWH14d, and EWH28d groups in ROS levels in both cerebral areas investigated (*P* > 0.05 for all groups; Fig. 6A-a).

Lipid peroxidation, in the same way, shows an effect of Cd (F _(1, 38)_ = 42.14, *P* < 0.0001), and an effect of the treatment with EWH (F _(2, 38)_ = 9.926, *P* = 0.0003) and there was no interaction between the two factors (*P* = 0.45).

Cd exposure increase significantly MDA levels in the cortex and striatum (t _(38)_ = 5.545, *P* < 0.0001, Fig. 6B; t _(38)_ = 5.853, *P* < 0.0001, Fig. 6b). Both EWH treatments reversed the lipid peroxidation to control levels in the cortex (Cd + EWHco: t _(38)_ = 3.697, *P* < 0.0103; EWHpre + Cd: F _(1, 38)_ = 3.272, *P* < 0.0341) and striatum (Cd + EWHco: t _(38)_ = 3.752, *P* < 0.0088; EWHpre + Cd: t _(38)_ = 4.177, *P* < 0.0030). There was no statistically significant difference between Ct, EWH14d, and EWH28d groups in MDA levels in the cortex or the striatum (*P* > 0.05 for all groups; Fig. 6B-b).

The antioxidant capacity did not show an effect of Cd (F _(1, 39)_ = 1.427, *P* = 0.23), and showed an effect of the treatment with EWH (F _(2, 39)_ = 7.357, *P* = 0.0019) and an interaction between the two factors (F _(2, 39)_ = 4.551, *P* = 0.0167).

The antioxidant capacity was reduced in the cortex (t _(39)_ = 3.371, *P* = 0.0255, Fig. 6C) and striatum (t _(39)_ = 5.161, *P* = 0.0001, Fig. 6C) on Cd exposure rats. EWH pretreatment (EWHpre + Cd: t _(39)_ = 1.950, *P* = 0.0003, Fig. 6D); EWH treatments had the effect of restoring antioxidant capacity on striatum (EWHpre + Cd: t _(39)_ = 8.429, *P* < 0.0001; Cd + EWHco: t _(39)_ = 6.067, *P* < 0.0001, Fig. 6c). Control situations did not differ from each other in the cortex or striatum (*P* > 0.05 for all groups; Fig. 6C-c).

SOD activity also was evaluated, and two-way ANOVA revealed an effect of Cd (F _(1, 30)_ = 43.44, *P* < 0.0001), and an effect of the treatment with EWH (F _(2, 30)_ = 6.193, *P* = 0.0056) and an interaction between factors (F _(2, 30)_ = 8.878, *P* = 0.0009).

The SOD activity did not differ between the control groups (*P* > 0.05). However, was reduced in Cd-exposure rats (t _(30)_ = 5.494, *P* = 0.0001, Fig. 6d) independently of EWH treatment in the striatum (EWHpre + Cd: t _(33)_ = 4.615, *P* = 0.0009; Cd + EWHco: t _(33)_ = 3.963, *P* = 0.0056). However, in the cortex, the reduction of SOD activity observed in the Cd group (t _(30)_ = 5.004, *P* = 0.0003) was restored only in EWH-pretreated rats (t _(30)_ = 4.455, *P* = 0.0004) (Fig. 6d). There was no statistically significant difference between Ct, EWH14d, and EWH28d groups in SOD activity in both cerebral areas investigated (*P* > 0.05 for all groups; Fig. 6D-d).

## Discussion

In this work, we demonstrated that Cd exposure is associated with decreased neurological and locomotor function in rats and that the consumption of an EWH treated with pepsin can both prevent (pretreatment with EWH) and avoid (cotreatment with EWH) partially this damage.

Cd is a toxic metal since from low to high exposure doses [46]. In addition, it is considered a widespread environmental pollutant, and the main source of toxic exposure is particulate inhalation by cigarette smoke [47], but it is also present in small amounts in foods [48]. Due to its presence in several products consumed by the general population, studies that report its toxicity are being performed. Furthermore, the literature has well described that Cd produces behavioral disorders and that these changes are related to its neurotoxic effect [47].

According to our best knowledge, for the first time, we demonstrated the neuromotor deficits caused by Cd through NDS evaluation in rats. Cd exposure is mainly related to brain and liver damage, which regulate neurological activities and energy metabolism, respectively [49]. Some authors suggest that Cd may cross the BBB and accumulate in the brain, damaging the central nervous system [50, 51], which could help to explain the observed deficits. On the other hand, the rats pre or cotreated with EWH did not present alterations in NDS. Consumption of EWH also decreased the number of falls in the RR test, demonstrating an improvement in motor coordination and balance.

However, the administration of EWH was not able to prevent or revert all types of deficits caused by Cd. Regarding the locomotor and exploratory activity of rats, assessed through the OF, the results showed that Cd induces locomotor and exploratory deficits and that both preexposure and cotreatment with EWH cannot prevent these deficits. A recent study using a dose curve of 1, 2, and 3 mg/g of Cd administered intraperitoneally demonstrated that the higher dose of Cd generates greater locomotor damage assessed in the OF, corroborating our results on the neurotoxic role and suggesting dose-dependent activity of Cd [47]. Exposure to high Cd doses has been previously related to increased oxidative stress in the reproductive and cardiovascular systems [22, 52]. It has been also reported that the accumulation of Cd in the nervous system generates an increase in reactive species [53], mainly superoxide anion [54], as occurs in other organs. Haider et al. [47] related locomotor damage to increased levels of malondialdehyde (MDA), a secondary product of lipid oxidation, and decreased levels of superoxide dismutase (SOD), an important antioxidant defense in the CNS.

In our study, increased levels of ROS and lipid peroxidation in the brain and a reduction in antioxidant defense were also observed. According to our results, high levels of lipid peroxidation were induced in the brain tissue of mice exposed to a low dose of Cd (1 and 2 mg/kg in drinking water after 30 days), and these findings were related to damage to antioxidants defense [55]. Acute exposure to a single dose similar to the one used in our study (1 mg/kg i.p.) also increased lipid peroxidation in the brain [56]. Chronic exposure to low concentrations of Cd (10, 25, and 50 mg/L in drinking water for 30 days) has also been reported to generate oxidant-antioxidant imbalance with damage at the GSH level and SOD, Catalase, and GPx activity [57]. In our findings, the scavenger capacity of EWH seems to have prevented the reduction of SOD activity by protecting neurological functions.

EWH, which has previously demonstrated neuroprotective properties associated with its antioxidant effect [24, 25], was used in our study with the intent to prevent or reverse motor deficits caused by Cd. However, although it was able to avoid the deficits measured by NDS and RR, we did not observe a reversal of the damage in the locomotor and exploratory activity in OF. These results partially agree with previous studies from our research group, which demonstrate that the administration of EWH does not alter the locomotor and exploratory capacity of animals that have been intoxicated with toxic metals, such as aluminum [25] or mercury [24].

Regarding the sensorial function, Cd-induced increase of the paw withdrawal on the HP task, which indicates deficits in the sensorial perception. Consumption of EWH was able to avoid this increase. A similar result has been recently described. Papp et al. [58] demonstrated that the somatosensory system was impaired after Cd exposure, even without detectable Cd deposition in the rat’s brain, and that these damages remained up to six weeks after exposure to Cd.

In the EPM task, we verified that Cd reduced the number of entries in the closed and open arms. Similarly, to our findings, behavioral damage induced by Cd-exposure has been reported at lower doses of exposure. Abdalla et al. [59] treated rats chronically with 2.5 mg/kg of Cd orally for 45 days and identified anxiolytic behavior in animals, relating this result to increased oxidative stress. Using the intraperitoneal route, Lamtai et al. [60] used a lower dose (0.25 and 0.5 mg/kg/day) and the same dose used in this study (1 mg/kg/day); however, twice of time of exposure (4 weeks), demonstrated that had lower entries in the open arms and exploratory behavior affected.

Different bioactive peptides derived from food proteins of different sources such as fish, soy, whey, milk, rice, silk, red algae, ginseng, and walnut have demonstrated neuroprotective capacity related to their anti-inflammatory, antioxidant, and/or anti-neurodegenerative properties, deserving to be investigated against neurological disorders [61]. However, there are currently very few studies that investigate the neuroprotective potential peptides derived from egg proteins, but some studies have shown promising results. A study carried out in healthy humans showed that dietary supplementation with an egg white lysozyme hydrolysate slightly improved the behavioral responses of the individuals. The authors associated this improvement with an increase in tryptophan levels and a consequent increase in the synthesis and release of serotonin in the CNS [62].

Egg white has naturally occurring low molecular weight (< 3 kDa) peptides with high antioxidant properties [63], and enzymatic hydrolysis of egg white proteins seems to strengthen this antioxidant effect, as observed in our study. Moreover, our EWH seems to have an essential benefit against metal intoxication. This functional food demonstrated an antioxidant effect against other metals reported by Rizzetti et al. [24], which prevented memory loss induced by exposure to low Hg concentration. EWH also prevented memory loss and cognitive deficits induced by exposure to low concentration (8.3 mg/kg for 60 days) and high concentration (100 mg/kg for 42 days) via drinking water from Al, as reported by Martinez et al. [25].

## Conclusions

Our findings showed that exposure to high Cd concentrations causes severe neurological damage with reduced exploratory capacity, motor dysfunctions, sensibility reduction, and induction of anxiety; on the other hand, pre or co-administration EWH can reduce oxidative stress and prevent partially the neurological damage induced by the metal. In conclusion, EWH could be used as a functional food with antioxidant properties capable of preventing neurological damage induced by Cd.

## Future Directions

Regarding the future development and commercialization of EWH as “functional food”, will be necessary to evaluate their bioactivity through intervention studies and clinical trials to ensure the effectiveness and safety of the product before its commercialization and more studies about bioavailability of isolated peptides and whole hydrolysates will be at the forefront of future functional food research. Another important aspect to highlight is that although there is growing interest in food-derived bioactive peptides, and it is relatively low cost to produce these peptides or their hydrolysates at laboratory scale, few researches aimed on the optimization process of food derived peptides and/or their hydrolysates, to obtain practical techniques used in food industry, making the leap from lab-scale to a commercial or pilot plant production because, in so many cases, it is too costly to produce them at industrial scale.
